# Independent factors associated with pneumonia among hospitalized patients with acute exacerbations of chronic obstructive pulmonary disease

**DOI:** 10.1097/MD.0000000000012844

**Published:** 2018-10-19

**Authors:** Songsong Yu, Qiuhong Fang, Yinjuan Li

**Affiliations:** a Department of Emergency, Beijing Shijitan Hospital; b Department of Respiratory and Critical Care Medicine, Beijing Chaoyang Hospital; c Department of Pulmonary and Critical Care, Beijing Shijitan Hospital, Capital Medical University, Beijing, China.

**Keywords:** acute exacerbation, chronic obstructive pulmonary disease, pneumonia, risk factors

## Abstract

Acute exacerbations (AE) affect the prognosis of hospitalized patients with chronic obstructive pulmonary disease (COPD). Pneumonia further affects their prognosis and early diagnosis of pneumonia in AECOPD is important to initiate treatments. This study aimed to examine the differences between hospitalized AECOPD patients with and without pneumonia in order to identify risk factors of pneumonia among hospitalized patients with AECOPD.

This was a retrospective case–control study of patients with COPD hospitalized at the respiratory ward of Beijing Shijitan Hospital, Capital Medical University, from October 2010 to October 2013. Patients were divided into the pneumonia and nonpneumonia groups based on exudations or opacities on chest computed tomography (CT) at admission. Data were analyzed using the chi-square test and independent 2-sample ANOVA in SPSS 20.0. Logistic regression analysis was used to identify the factors independently associated with pneumonia. *P < *.05 was considered statistically significant.

A total of 164 patients were included. Smoking history (OR = 2.646, 95%CI 1.153–6.074, *P = *.022), use of drugs during the stable stage (OR = 0.435, 95%CI 0.216–0.877, *P = *.020), D-dimer levels (OR = 1.001, 95%CI 1.000–1.002, *P = *.049), percentage of neutrophils (OR = 0.271, 95%CI 0.078–0.940, *P = *.040), and magnitude of neutrophils increase (OR = 0.946, 95%CI 0.896–0.999, *P = *.046) were independently associated with pneumonia in patients with AECOPD. For severe and very severe COPD patients, smoking history (OR = 4.426, 95%CI 1.458–13.435, *P = *.009), use of drugs during the stable stage (OR = 0.384, 95%CI 0.168–0.877, *P = *.042), and fever (OR = 0.426, 95%CI 0.187–0.969, *P = *.023) were independently associated with pneumonia.

Smoking history, use of drugs during the stable stage, and percentage of neutrophils are independently associated with CT-diagnosed pneumonia among hospitalized AECOPD patients.

## 1 Introduction

The main characteristic of chronic obstructive pulmonary disease (COPD) is persistent airflow limitation that progresses with disease course. COPD accounts for the third cause of hospitalization worldwide and the fourth cause of mortality.^[1]^ Acute exacerbation of COPD (AECOPD) is the main cause of hospitalization due to COPD, and most AECOPD events are due to bacterial or viral infection.^[2]^ Pneumonia is one of the most common causes of hospitalization among patients with AECOPD, and about one-third of AECOPD patients show pulmonary exudation and consolidation at imaging.^[3,4]^

Even if it has been shown that pneumonia is among the most common causes of hospitalization of patients with COPD, the exact nature of pneumonia in relation to AECOPD is still controversial. Indeed, it has been suggested that pneumonia in COPD patients should be regarded as AECOPD events.^[5]^ In addition, an audit of hospital admission for AECOPD by the British Thoracic Society included patients with changes consistent with pneumonia on chest x-ray.^[6]^ Some authors consider that the clinical manifestations of patients with COPD and pneumonia are in accordance with the clinical diagnosis of AECOPD, so pneumonia is not considered as an exclusion criteria of AECOPD.^[7]^ Nevertheless, chest x-ray is not sensitive enough for identifying pulmonary exudation and consolidation; indeed, more detailed imaging can reveal consolidation in patients with initially negative chest x-ray.^[8]^ Lieberman et al^[5]^ showed that the sociodemographic characteristics and severity of the underlying COPD were similar between patients with nonpneumonic AECOPD and those with coexistent chest x-ray consolidation, but the later had more abnormal acute clinical and physiological markers. These results suggested that coexisting consolidation could identify patients with a more severe acute illness, but not necessarily a different disease process.

Early diagnosis and treatment initiation play important roles in the prognosis of patients with AECOPD and pneumonia, identifying risk factors and markers for pneumonia in patients with AECOPD could help initiate the proper treatments as early as possible and improve their prognosis. Braeken et al^[9]^ showed that age, female gender, and smoking were associated with the risk of pneumonia in COPD. Kurashima et al^[10]^ showed that low body mass index, emphysema, and absence of pneumococcal vaccination increased the risk of pneumonia in COPD. Other risk factors also include comorbid cardiovascular diseases, dementia, prior severe AECOPD events, home oxygen, and inhaled corticosteroids.^[11–14]^

Nevertheless, different risk factors could be observed among different populations.^[15]^ Therefore, the present study aimed to analyze the differences between hospitalized AECOPD patients with and without pneumonia (as determined by CT) in order to identify risk factors of pneumonia among patients hospitalized for AECOPD.

## 2 Materials and methods

### 2.1 Study design

This was a retrospective case-control study of patients with COPD hospitalized at the respiratory ward of Beijing Shijitan Hospital, Capital Medical University, from October 2010 to October 2013. This study was approved by the institutional review board of Beijing Shijitan Hospital, Capital Medical University (approval number 2016-22). As the study was retrospective and entirely based on the medical charts, the need for individual consent was waived by the committee because there was strictly no contact with the patients. In addition, the data were de-identified.

### 2.2 Patients

The diagnoses of COPD and AECOPD were defined according to the standards of the Chinese Thoracic Society (revised in 2007).^[16]^ The severity of the disease was classified into mild, moderate, severe, and very severe, based on the same standards.^[16]^ The diagnosis of pneumonia was in accordance to pulmonary exudation and consolidation on chest computed tomography (CT) scan at admission. The CT scans were routinely performed and interpreted by 2 radiologists according to routine criteria.^[17]^ The findings for CT scans included (but were not limited to) ground-glass attenuation, thickening of interlobular septa, airspace consolidation, traction bronchiectasis, honeycombing, intralobular linear opacities, and centrilobular nodules.^[18]^

The inclusion criterion was: diagnosis of AECOPD. The exclusion criteria were: pulmonary edema, pulmonary hemorrhage, pulmonary fibrosis, tuberculosis, lung cancer, pulmonary embolism, or bronchiectasis; or incomplete clinical data of the AECOPD events. Patients were followed-up by telephone or outpatient visit after discharge. The last follow-up was in October 2014.

The eligible population included all patients hospitalized for AECOPD during the study period. The evaluable population included all patients hospitalized for AECOPD during the study period and meeting inclusion/exclusion criteria. The per-protocol population included the patients of the evaluable population and with available follow-up after hospitalization.

### 2.3 Data collection

Data including gender, age, height, body weight, body mass index (BMI) (kg/m^2^), smoking history, comorbidities, arterial blood gas analysis, lung function during the stable stage (no cough, expectoration, or asthmatic syndrome),^[16]^ drugs during the stable stage before the exacerbation event that led to hospitalization, antibiotics during hospitalization, blood urea nitrogen (BUN), serum creatinine (CRE), blood uric acid (UA), D-dimer levels, white blood cell count, neutrophil, erythrocyte sedimentation rate (ESR), C-reactive protein (CRP), forced expiratory volume in 1 second (FEV1)/full vital capacity (FVC) ratio, hospital stay, and outcomes (hospitalization and death) were extracted from the medical charts. COPD was graded:^[19]^ FEV1/FVC <70% and FEV1 >80%, mild; FEV1 50%–80%, moderate; FEV1 30%–49%, severe; FEV1 <30% or FEV1 30%–50% combined with chronic respiratory failure, very severe.

### 2.4 Statistical analysis

Data were analyzed using SPSS 20.0 (IBM, Armonk, NY). Continuous variables were shown as mean ± standard deviation. Categorical variables were presented as absolute numbers and proportions. Differences between the 2 groups were analyzed using the chi-square test for categorical variables and the independent 2-sample analysis of variance for continuous variables. Factors independently associated with AECOPD with pneumonia and with recurrence of AECOPD were identified by logistic regression analysis (methods: backward, Wald). To limit the number of events-per-variable, variables with a *P*-value <.1 in logistic regression analyses were included in the logistic regression model. Two-sided *P*-values <0.05 were considered to be statistically significant.

## 3 Results

### 3.1 Characteristics of the patients

**Figure 1:**
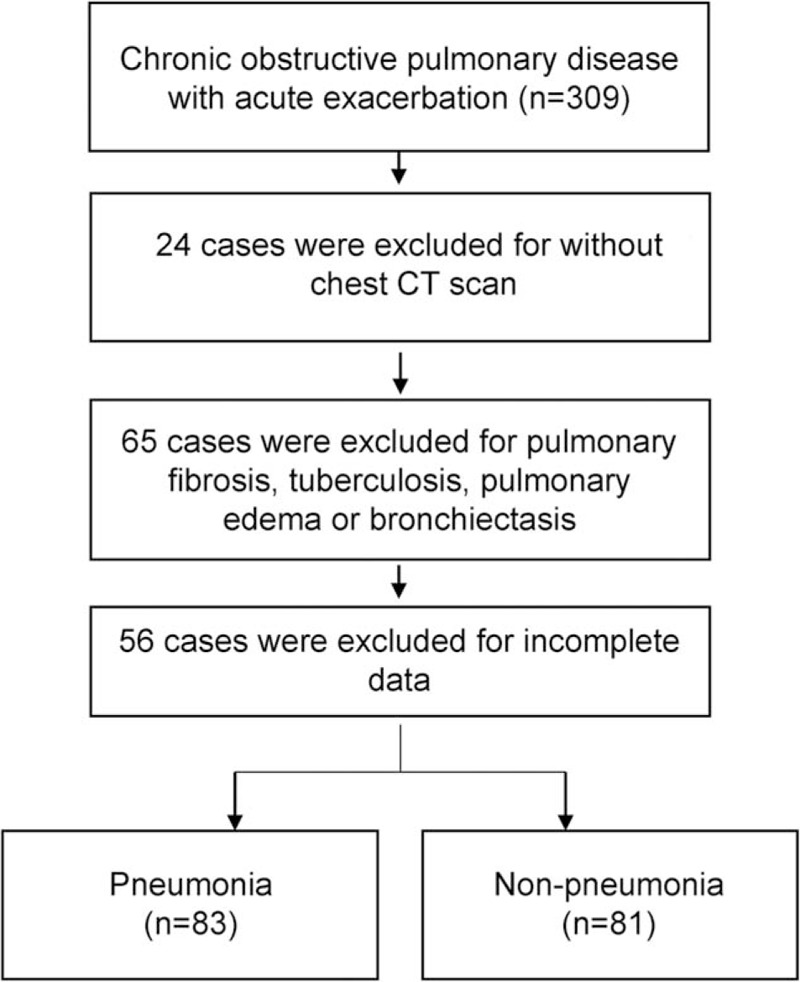
Study flowchart.

A total of 309 patients hospitalized for AECOPD were reviewed (eligible population). Among them, we excluded 24 patients with missing chest CT scan, 65 with pulmonary edema, pulmonary fibrosis, tuberculosis or bronchiectasis on chest CT scan, and 56 with incomplete AECOPD data. Finally, 164 patients with AECOPD were included for analysis: 83 (50.6%) patients with pneumonia and 81 (49.4%) without (evaluable population) (Fig. 1).

Among the 164 patients, mean age was 74.1 ± 8.6; 69.5% of the patients were men; the average BMI was 23.9 ± 4.3, and the mean hospital stay was 10.5 ± 7.4 days. There were significant differences in age (*P = *.027), smoking history (*P = *.049), D-dimer levels (*P = *.030), and duration of hospitalization (*P = *.039) between the 2 groups (Tables 1–3).

**Table 1:**
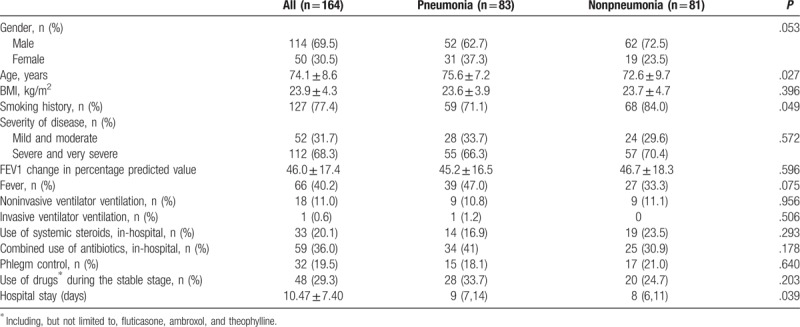
Clinical data between the pneumonia and nonpneumonia groups.

**Table 2:**
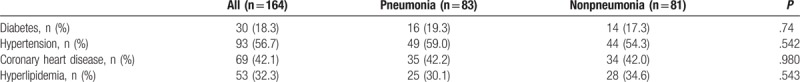
Comorbidities between the pneumonia and nonpneumonia groups.

**Table 3:**
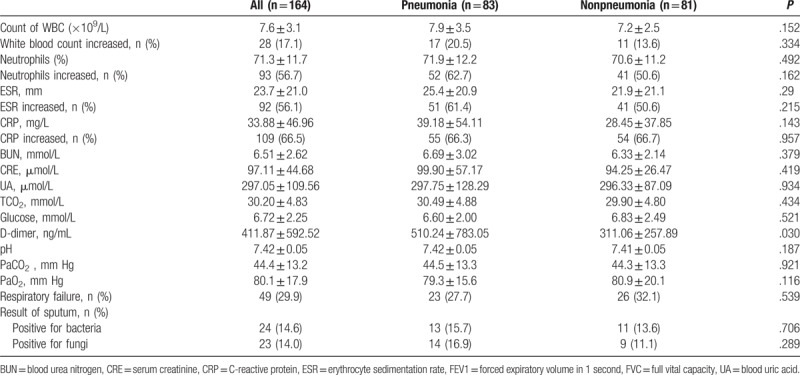
Laboratory results between the pneumonia and nonpneumonia groups.

### 3.2 Multivariable analysis

Multivariable logistic regression analysis showed that smoking history (OR = 2.646, 95%CI 1.153–6.074, *P = *.022), use of drugs (including, but not limited to, fluticasone, ambroxol, and theophylline) during the stable stage (OR = 0.435, 95%CI 0.216–0.877, *P = *.020), D-dimer levels (OR = 1.001, 95%CI 1.000–1.002, *P = *.049), percentage of neutrophils (OR = 0.271, 95%CI 0.078–0.940, *P = *.040), and magnitude of neutrophils increase (OR = 0.946, 95%CI 0.896–0.999, *P = *.049) were independently associated with pneumonia in patients with AECOPD (Table 4).

**Table 4:**
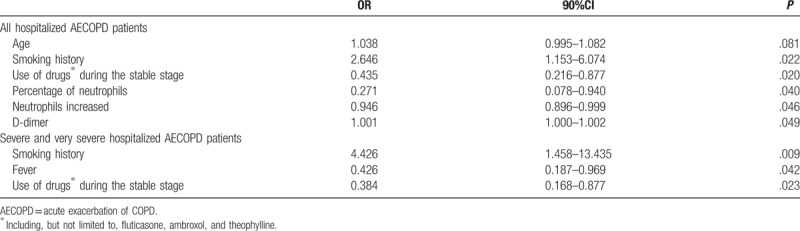
Risk factors of pneumonia for hospitalized AECOPD patients.

For severe and very severe COPD patients, smoking history (OR = 4.426, 95%CI 1.458–13.435, *P = *.009), use of drugs (including, but not limited to, fluticasone, ambroxol, and theophylline) during the stable stage (OR = 0.384, 95%CI 0.168–0.877, *P = *.042), and fever (OR = 0.426, 95%CI 0.187–0.969, *P = *.023) were independently associated with pneumonia (Table 4).

### 3.3 Prognosis

No significant differences were observed in clinical AECOPD recurrence (47.0% vs 39.5%, *P = *.334), pneumonia occurrence/reoccurrence (36.1% vs 34.6%, *P = *.833), and mortality (4.8% vs 1.2%, *P = *.378) between the 2 groups during the 1-year follow-up.

## 4 Discussion

COPD is a preventable and treatable airflow limitation disease, and AECOPD is an important reason for poor prognosis.^[7]^ The majority of AECOPD events are attributed to bacterial or viral infection.^[2]^ Pneumonia is a common cause of hospitalization for patients with COPD,^[20]^ and pneumonia is considered a serious condition in patients with COPD because pneumonia can be considered as a more disseminated or invasive disease (affecting alveoli and bronchioles) than AECOPD, which is characterized by inflammation that normally affects only the bronchi.^[21]^ Identifying pneumonia in patients with COPD is important to initiate early treatments. Therefore, this study aimed to examine the differences between hospitalized AECOPD patients with and without pneumonia (diagnosed by CT) in order to identify the factors associated with pneumonia. The results showed that smoking history, use of drugs (including, but not limited to, fluticasone, ambroxol, and theophylline) in the stable stage, and percentage of neutrophils are independently associated with pneumonia for hospitalized patients with AECOPD.

In the present study, the frequency of pneumonia in patients hospitalized for AECOPD was 50.6%, which was similar to a study by Merino-Sánchez et al,^[22]^ but higher than that reported by others.^[3,23]^ The reason for the high pneumonia ratio may be the usage of chest CT, since chest CT has a higher diagnostic power for pneumonia that plain x-ray. AECOPD patients with pneumonia were significantly older and had been hospitalized longer than patients without pneumonia. This suggests that elderly patients with COPD were more susceptible to pneumonia during acute exacerbations, and patients with pneumonia were hospitalized for significantly longer duration, which were consistent with previous reports.^[23–25]^ Contrary to previous reports,^[23,26,27]^ patients with long-term use of inhaled corticosteroids in the stable stage were not found to be more susceptible to pneumonia. Discrepancies can be due to a number of reasons, including (but not limited to) the severity of disease (in the present study, mainly severe and very severe COPD), genetics, pollution, life habits, and financial status (not everyone has medical insurance in China).

In the present study, the proportion of smoking history in the nonpneumonia group was significantly higher than in the pneumonia group (84%). In addition, females represented a higher proportion of the patients who had no smoking history but exposure to biofuels, and both smoking and biofuels show similar substantial toxicity effects on the airways.^[28]^ Therefore, patients in the nonpneumonia group typically suffered from bronchitis, while patients in the pneumonia group had alveolar exudation.

In the present study, smoking history and D-dimer levels were independently associated with pneumonia among hospitalized AECOPD patients. Lippi et al^[29]^ suggested that infection was the most frequent diagnosis for patients with elevated D-dimer levels in the emergency room. Nastasijević Borovac et al^[30]^ indicated that plasma D-dimer levels correlated better than standard inflammatory markers with the severity of disease and risk of mortality in patients with CAP. Accordingly, we found that D-dimer levels were independently associated with pneumonia of hospitalized AECOPD. D-dimer levels are also associated with the risk of deep vein thrombosis and pulmonary embolism,^[31,32]^ but such events were not observed in the present study.

On the other hand, the use of drugs during the stable stage before AECOPD, percentage of neutrophils, and increased neutrophils were protective factors for pneumonia, partially supported by a previous study.^[3]^ Nevertheless, drugs being a protective factor for pneumonia is surprising as the use of ICS is known to alter the patient's airway microenvironment, increasing the risk of bacterial and fungal infections.^[33–37]^ The reason for this discrepancy is currently unknown.

Treatments during the stable stage included theophylline, phlegm control, and inhaled bronchial LAMA. It is known that inhaled corticosteroids increase the risk of pneumonia in patients with COPD.^[38]^ Neutrophil count is known to be a marker of bacterial infection, including pneumonia.^[39,40]^ AECOPD events are associated with such infections, and exacerbation events caused by bacteria are associated with longer hospital stays and more frequent exacerbations.^[41–43]^ When the percentages of neutrophils are increased, clinicians tend to use antibiotics in the initial stage of AECOPD, which could restrain the inflammation of bronchial and pulmonary alveoli effectively, and then reduce the exudation and consolidation on the chest CT scan. Early use of antibiotics can delay or deter the infection progression, so it became a protective factor. Nevertheless, the timing of blood sampling can affect the neutrophil results and this should be considered as an indicator of an increased risk of the presence of pneumonia, and not as a diagnostic factor.

In this study, there was no association between the severity of COPD and pneumonia, which was in contrast to a previous report.^[44]^ We determined pneumonia using chest CT, which is more sensitive than chest x-ray, and can easily detect early pneumonia. The proportion of patients with severe and very severe COPD was up to 68.3% (112/164) in this study, which was higher than in previous studies.^[24,25]^ Nevertheless, for severe and very severe COPD patients, smoking history, fever, and use of drugs (including, but not limited to, fluticasone, ambroxol, and theophylline) during the stable stage were independently associated with pneumonia among hospitalized AECOPD patients. This was consistent with a previous study.^[24]^ In the elderly hospitalized AECOPD patients, pneumonia should be suspected among those with previous smoking history and severe COPD.

Even though it was not part of the main objective, the present study examined the follow-up of the patients with AECOPD. Previous studies showed that the 90-day mortality of AECOPD patients with pneumonia was significantly higher than that of those without pneumonia, suggesting that patients with pneumonia had a worse prognosis.^[24,25]^ Nevertheless, this observation is controversial and Huerta et al^[45]^ suggest that pneumonic and nonpneumonic AECOPD have similar short- and long-term outcomes. In the present study, hospitalized patients with AECOPD and pneumonia had slightly higher (but not significant) rates of recurrence and mortality during the 1-year follow-up than patients without pneumonia. Nevertheless, there have been very few recent studies on hospitalized AECOPD patients with pneumonia with a 1-year follow-up and additional studies are needed to confirm these findings.

This study is not without limitations. First, this was a retrospective study (limiting the data to what was available in the medical charts), the sample size was small, the patients were from a single center, and the patients were from a short time period (October 2010–October 2013). Multicenter prospective cohort studies should be performed. Second, during data collection, some patients were excluded because of missing data, which could introduce a bias. Nevertheless, we have to assume that the distribution of missing data is random, minimizing this bias. Third, we could not examine the causative agents because of the small sample (leading to too small subgroups) and because the exact causative agent could not be determine in some cases. These cases could be due to viruses, but identifying viruses is difficult and many physicians do not push the matter since there is no treatment in most cases. Nevertheless, this issue is important since patients with different causative agents could have different epidemiology and outcomes.^[46]^ Finally, the patients received different ICS and treatments, which in itself could lead to bias because of different potency, regimens, etc. Future studies should examine closely the drugs taken by the patients, but a larger sample size could be necessary to reach some statistical power. Thus, to generalize and confirm our findings, a multicenter prospective study with larger numbers of patients is needed.

## 5 Conclusions

Smoking history, use of drugs (including, but not limited to, fluticasone, ambroxol, and theophylline) during the stable stage, and percentage of neutrophils are independent risk factors of CT-diagnosed pneumonia for hospitalized AECOPD patients. For severe and very severe COPD, smoking history, use of drugs during the stable stage, and fever were independent risk factors for pneumonia. These results suggest factors that could help detect pneumonia in patients with AECOPD in order to initiate early treatments.

## Author contributions

**Conceptualization:** Qiuhong Fang.

**Data curation:** Songsong Yu, Yinjuan Li.

**Formal analysis:** Songsong Yu, Yinjuan Li.

**Investigation:** Yinjuan Li.

**Methodology:** Qiuhong Fang, Songsong Yu.

**Supervision:** Yinjuan Li.

**Writing – original draft:** Songsong Yu.

**Writing – review & editing:** Qiuhong Fang.
